# BK Polyomavirus Virus Glomerular Tropism: Implications for Virus Reactivation from Latency and Amplification during Immunosuppression

**DOI:** 10.3390/jcm8091477

**Published:** 2019-09-17

**Authors:** Donald J. Alcendor

**Affiliations:** Center for AIDS Health Disparities Research, Meharry Medical College, 1005 Dr. D.B. Todd Jr. Blvd., Hubbard Hospital, 5th Floor, Rm. 5025, Nashville, TN 37208, USA; dalcendor@mmc.edu

**Keywords:** polyomavirus, BKV, kidney, glomerulus, BKVAN, nephropathy, transplantation

## Abstract

BK polyomavirus (BKPyV), or BKV infection, is ubiquitous and usually non-pathogenic, with subclinical infections in 80–90% of adults worldwide. BKV infection is often associated with pathology in immunocompromised individuals. BKV infection often is associated with renal impairment, including ureteral stenosis, hemorrhagic cystitis, and nephropathy. BKV infection is less commonly associated with pneumonitis, retinitis, liver disease, and meningoencephalitis. BKV is known to replicate, establish latency, undergo reactivation, and induce clinical pathology in renal tubular epithelial cells. However, recent in vitro studies support the notion that BKV has expanded tropism-targeting glomerular parenchymal cells of the human kidney, which could impact glomerular function, enhance inflammation, and serve as viral reservoirs for reactivation from latency during immunosuppression. The implications of BKV expanded tropism in the glomerulus, and how specific host and viral factors that would contribute to glomerular inflammation, cytolysis, and renal fibrosis are related to BKV associated nephropathy (BKVAN), have not been explored. The pathogenesis of BKV in human glomerular parenchymal cells is poorly understood. In this review, I examine target cell populations for BKV infectivity in the human glomerulus. Specifically, I explore the implications of BKV expanded tropism in the glomerulus with regard viral entry, replication, and dissemination via cell types exposed to BKV trafficking in glomerulus. I also describe cellular targets shown to be permissive in vitro and in vivo for BKV infection and lytic replication, the potential role that glomerular parenchymal cells play in BKV latency and/or reactivation after immunosuppression, and the rare occurrence of BKV pathology in glomerular parenchymal cells in patients with BKVAN.

## 1. Introduction 

BK polyomavirus (BKPyV, hereafter referred to as BKV) is a member of the genus *Betapolyomavirus*, which belongs to the Polyomaviridae family of viruses that includes JC polyomavirus, or JCPyV, and Simian-virus 40 (SV40 virus) [1,2,3,4,5,6]. BKV was first isolated by Gardner in 1971, from the urine sample of a renal transplant patient diagnosed with ureteral stenosis with the initials “B.K.” [7]. BKV is a small non-enveloped, icosahedral, circular, doubled-stranded DNA virus that is 40–45 nm in diameter, with a genome size of approximately 5 kb (kilobases) [8,9]. BKV was first reported in 1995 as being a cause of allograft failure in renal transplant patients [10]. It is now recognized as an emerging pathogen in renal transplant patients, with increased incidence that correlated with the use of more potent iatrogenic immunesuppressants such as tacrolimus (FK 506) and mycophenolate mofetil (Cellcept) [11,12,13,14,15,16].

While primary infection with BKV is usually asymptomatic and occurs early in life with a seroprevalence of 80–90% in adults worldwide, it is often associated with pathology in immunocompromised individuals [17,18]. BKV has a seroprevalence of 65% to 90% in children aged 5–9 years, and can be transmitted via respiratory, uro-oral, and feco-oral borne routes [19,20]. Because latent BKV is known to reactivate in patients who have immunocompromised incidence of conditions associated with BKV infection such as encephalitis, nephritis, hemorrhagic cystitis, retinitis, and pneumonia, it has also been reported in HIV-1 infected patients [21,22]. HIV-1 patients also experience a higher prevalence of BKV viruria than healthy individuals that show a positive correlation with the degree of immunosuppression [23]. 

BKV reactivation after immunosuppression in transplant recipients can result in clinical disease in the form of BKV associated nephropathy (BKVAN), leading to ureteral stenosis, tubular interstitial damage, as well as hemorrhagic cystitis in bone marrow transplant patients [24,25,26]. Primary BKV infection is accompanied by viral replication, followed by the establishment of latency in renal tissue [27]. BKV-associated pathology linked to immunosuppression includes diseases of the respiratory tract, urinary bladder, kidney, the central nervous system (CNS), eye, digestive tract, and endothelium [20]. BKV reactivation from latency is followed by viruria, which occurs in up to 20% of asymptomatic immunocompetent individuals, and in 20–60% of immunocompromised patients [28]. Approximately 80% of renal transplant recipients experience BK viruria and among those 5–10% develop BKVAN [28]. Virus infection leading to viremia, interstitial inflammation, graft rejection with the progression of interstitial fibrosis, and tubular atrophy, can lead to allograft failure and end stage renal disease (ESRD). ESRD represents an important health disparity among underserved populations [29,30,31,32]. Currently, there is no specific treatment for BKVAN. With no effective consistent antiviral therapy, pre-emptive reduction of maintenance immunosuppression and/or changes to the immunosuppressive regimen is recommended to control BKV replication, which may lead to an increased risk of allograft rejection [27]. The underlying mechanisms and kinetics of BKV infection in BKVAN remain largely unexplored. Primary infection of glomerular parenchymal cells could lead to progressive inflammation, injury, and cytolysis, which contribute to renal fibrosis and likely lead to ESRD. 

## 2. BKV Infection and Post-Transplant Kidney Disease

In the adult population, there is a high prevalence of BKV infection and latency in renal tissue that usually remains asymptomatic in immunocompetent individuals, but predisposes renal transplant patients that require immunosuppression to BKV reactivation and replication. Approximately 50–80% of patients that develop BKVAN also experience graft failure [33]. The incidence of graft failure is dependent on the degree of glomerular inflammation caused by proinflammatory cytokines, the influx of immune effector cells, BKV lytic replication, and lysis of renal tubular epithelial cells that can lead renal fibrosis and subsequent graft failure [34,35,36]. In renal transplant patients, reactivation of BKV occurs in the graft and the infection is donor-derived [37], with higher rates of reactivation occurring with donors that are BKV seropositive [37]. BKV reactivation after renal transplantation is usually first observed by the appearance of virus-infected uroepithelial cells, known as decoy cells, that are found in the urine or BKV DNA in the urine, which is followed by a viremic phase that occurs approximately one month post-transplantation, according to Hirsch et al. [38]. BKV viremia precedes BKVAN. It is a better predictor of pathology associated with nephropathy than viruria, especially when accompanied by viral titers >10,000 copies/mL [39,40]. The timing of BKV reactivation and replication after transplant has been associated with several factors. These include the intensity of the immunosuppressive regimen involving the use of tacrolimus or mycophenolate mofetil, recipient-related factors (such as patient age, male sex, non-African American race), donor-related factors (such the degree of HLA mismatches, BKV seropositivity), and viral-related factors (such as the BKV genotype) [27,41]. In addition, other factors, such as renal injury associated with variation in cold ischemia time, delayed allograft function, and the placement of ureteral stents, have also been reported to influence BKV reactivation [42,43]. However, conclusive diagnosis of BKVAN requires the detection of viral inclusion bodies on renal biopsies, as well as confirmation of genome detection by in situ hybridization or viral antigen detection via immunohistochemical staining for the BKV large T antigen (LTAg) [44]. The BKV LTAg is known to cross-react with antibodies against the LTAg of simian virus 40 (SV40) that shares 70% genome sequence homology with BKV. While ultrastructural analysis by electron microscopy is highly sensitive for detecting BKV, and has been used to diagnose BKV infection, the presence of BKV alone may not be sufficient to confirm a BKVAN diagnosis. The reliability of these techniques varies due to non-specific binding of immunoglobulins and DNA oligomers in human tissue, hence, standardization is warranted. 

BKVAN is divided to three histopathological grades: A, B, and C. Grade A BKVAN presents as inflammation in the tubular epithelial with the absence of tubular epithelial necrosis. Grade B BKVAN is defined as more progressive in pathology, involving both tubular epithelial cell necrosis as well as tubular epithelial cell lysis. Grade C BKVAN is defined as the presence of interstitial fibrosis that can ultimately lead to ESRD [45]. A strong correlation exists between graft survival based on histopathological grades of BKVAN, with Grade A having the best prognosis for graft survival at two years (90%) and Grade C having the worst (50%) [46]. 

Histological lesions in BKVAN are normally scored by the Banff 97 classification of renal allograft pathology to indicate severity [46,47,48]. Several biomarkers have been examined to predict the onset of BKVAN and the relationship to graft failure, which includes urine analysis by PCR amplification of BKV-VP1, or the presence of grandzyme B, proteinase inhibitor-9, plasminogen activator inhibitor-1, as well as the urine polyomavirus Haufen Test to determine the presence of urinary cast [49,50,51,52,53,54,55,56]. There is currently no specific universal screening biomarker that is widely used in clinical practice that consistently predicts the early onset of BKVAN and correlates strongly with graft survival. Furthermore, the characteristic changes reported for BKVAN-associated renal pathology may only exist in a fraction of infected patients in varying degrees. 

**Figure 1:**
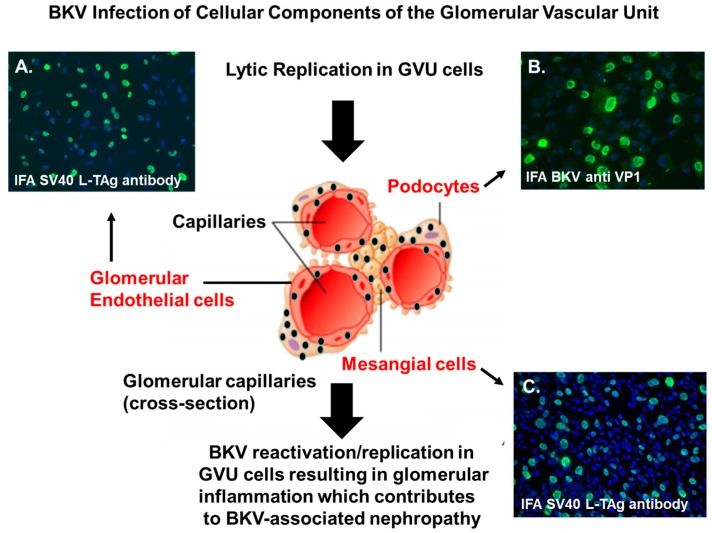
BK polyomavirus (BKV) infection of GVU cells. Immunofluorescent staining of GVU cells infected with BKV. (**A**) Primary human glomerular endothelial cells infected with BKV for 96 h and stained with a monoclonal antibodies against the SV40 Large T antigen (LTAg). (**B**) Human podocytes infected with BKV for 96 h and stained with monoclonal antibodies against the BKV major capsid protein VP1. (**C**) Primary human mesangial cells infected by BKV for 96 h and stained with a monoclonal antibody targeting the SV40 (LTAg). Nuclei were stained blue with 4′,6-diamidino-2-phenylindole (DAPI). All images were obtained using a Nikon TE2000S microscope mounted with a charge-coupled device (CCD) camera at ×200 magnification.

Expanded BKV tropism for glomerular parenchymal cells or GVU cells that includes glomerular podocytes, mesangial cells, and glomerular endothelial cells, has been confirmed by vitro studies in my laboratory [34] (Figure 1). This finding will require further investigation.

## 3. BKV Entry and Dissemination in the Glomerulus and the Cell Types Exposed to BKV Trafficking

**Figure 2:**
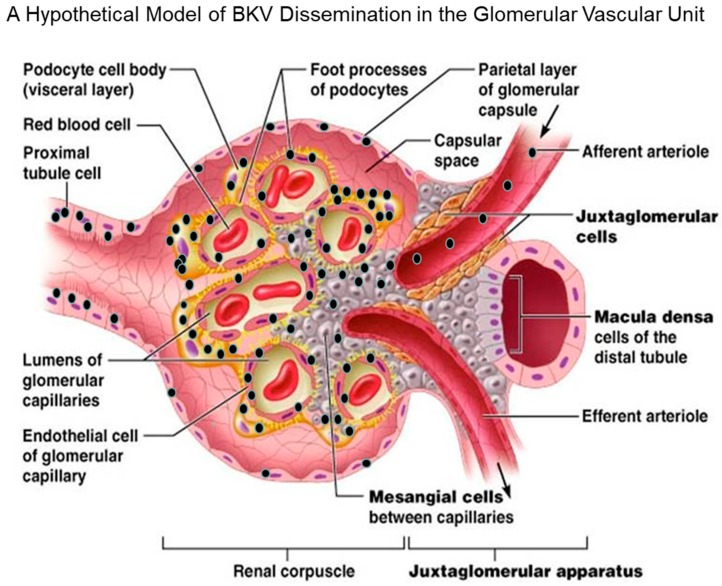
A hypothetical mode for BKV dissemination in the glomerular that includes GVU cells. BKV (black spheres) enters the glomerulus of the renal compartment via the afferent arteriole during the viremic phase of infection. This leads to the initial infection of GVU cells, namely the mesangial cells. Next, the virus spreads from mesangial cells to glomerular podocytes, and then locally to glomerular endothelial cells that are also highly permissive for infection in vitro and reported to be infected by BKV in vivo. The virus then encounters the parietal cells of the glomerular capsule that are reported to be permissive for BKV in vivo. Finally, the virus further disseminates and infects the proximal tubular epithelial cells that are highly permissive for BKV infection in vitro and in vivo. Widespread virus infection and replication in GVU targets cells, along with tubular epithelial cells and parietal glomerular capsular cells, would theoretically contributes to the viruria, viremia, inflammation, and nephropathy. Model of BKV entry and existence in the glomerulus (modified with permission from *Pearson Education Inc.* 2013 (unpublished data)).

In a hypothetical model proposed by Popik et al., BKV enters the glomerular parenchyma via the afferent arteriole during the viremic phase of infection, leading to viral dissemination and the initial exposure of glomerular mesangial cells to the virus (Figure 2). The virus then spreads from the mesangial cells to the glomerular podocytes and endothelial cells of glomerular capillaries. BKV may spread first to the parietal cells of the glomerular capsule and then to the proximal tubular cells before appearing in urine. The initial and continual dissemination track of BKV would also be influenced by the turbulence produced by blood flow and renal filtration. Most recently, a report by Popik et al. suggests that the tropism of BKV in the human kidney involves glomerular parenchymal cells, which have been shown to be permissive for BKV in vitro [34]. The potential role of these cells in viral latency, viral reactivation, viral load, viremic conversion, and BKVAN-associated renal pathology is unknown. 

## 4. Cellular Targets that are Permissive for BKV Infection and Lytic Replication 

### 4.1. Tubular Epithelial Cells

A comprehensive examination of cellular targets for BKV infectivity in the proximal and distal glomerular compartments of the human kidney has not been reported. Rather, the focus of BKV infectivity and pathogenesis has been mainly on tubular epithelial cells, and most studies have proposed them as the primary viral reservoir and main driver of pathogenic pathways that lead to fibrosis in BKVAN [57,58]. These reports conclude that renal tubular epithelial cells are the major sites of viral persistence and reactivation in immunosuppressed kidney transplant patients [59]. Tubular epithelial cells are important in vitro and in vivo targets for BKV infection and replication. Findings from several studies support the notion that tubular epithelial cell infection, dysfunction, necrosis, and death are essential prerequisites for renal fibrosis associated with BKVAN. However, studies by de Kort H et al. suggest that rapid lytic replication of BKV occurs in tubular epithelial cells, because these cells are immunologically tolerant to BKV infection rendering them more susceptible to high levels of lytic replication when compared to other glomerular cells that are more immunologically responsive to BKV infection, as demonstrated by a robust induction of interferon beta (IFNβ) and CXCL10 in the latter, post-infection [60]. 

### 4.2. Bowman’s Capsular Epithelial Cells (BCEC)

By examining renal biopsies from renal transplant patients with BKVAN, Celik and Randhawa detected cytopathic effects of BKV in Bowman’s capsular epithelial cells (BCECs) at the parietal layer of Bowman’s capsule [61]. The authors observed BKV cytopathology in BCECs in 36/124 biopsies (29%) from 83 patients examined with BKVAN in the allograft kidney, using H&E stained-light microscopy and immunohistochemistry [61]. The authors used in situ hybridization to confirm the presence of BKV DNA in BCECs [61]. Moreover, they also found that BKV cytopathology in BCECs correlated with high viral loads in the tubular epithelium [61]. Interestingly, tubular epithelial cells that are highly permissive for BKV lytic replication share the same embryologic origin as BCECs. Therefore, it is reasonable to speculate that BCECs are also permissive for BKV. However, the role for BCECs in BKV latency and reactivation is currently unknown. Comprehensive in vivo and in vitro studies of BCECs are warranted. The role of BCECs in viral latency and reactivation has not been explored. Results from studies that examine renal biopsies from transplant patients with BKVAN suggest that BKV infection of BCECs is rare. Nonetheless, it would be interesting to determine if BCECs play a similar role to that of tubular epithelial cells in BKVAN, due to their common origin. 

### 4.3. Mesangial Cells

Until recently, there were no reports of BKV infection of mesangial cells. A study published in 2019 by Popik et al., shows that primary human renal mesangial cells are permissive for BKV infection in vitro. Specifically, the authors found that mesangial cells expressed BKV late genes 96 h post-infection, without exhibiting evidence of cytopathology [34]. However, immunofluorescent staining revealed high levels of virus replication in these cells, as demonstrated by nuclear staining of BKV-infected cells with an antibody against the SV40 LTAg, along with high levels of VP1 transcription [34]. The authors also observed significant induction of CXCL10 and IFNβ expression in BKV-infected cells that correlated with increased virus replication over a time course of infection. However, it is currently unclear if mesangial cells play a role in BKVAN progression in vivo. There are currently no reports of BKV-infected mesangial in biopsies from renal allograft patients with BKVAN. In a study by Celik et al., they report immune complex deposition in the mesangium and an increased mesangial cell matrix in renal biopsies from patients with BKVAN. However, the authors did not observe evidence of BKV infection in mesangial cells [61]. Since mesangial cells are immunologically responsive to BKV infection, as evidenced by induction of CXCL10 and IFNβ [34,35], they may be more effective at viral clearance than tubular epithelial cells. In addition, there could be host factors in the glomerular microenvironment induced in mesangial cells after infection that render them less permissive for BKV infection in vivo. 

### 4.4. Glomerular Podocytes

Currently, there is only one report, by Brealey, describing a case study of BKVAN that shows evidence of viral particles in glomerular subepithelial humps. The author used transmission electron microscopy to analyze the glomeruli in a renal biopsy from a 59-year-old female kidney transplant patient who was experiencing symptoms of graft rejection [62]. There was clear clinical evidence from the examination of biopsy tissue to support a diagnosis of immune complex glomerulonephritis. Virus particles were observed in deposits in the cytoplasm of podocytes [62]. The authors confirmed the diagnosis of BKVAN by immunoperoxidase staining using BKV- specific antibodies. The author also observed evidence of cytoplasmic clearance of BKV by podocytes from the glomerular basement membrane [62]. However, this case study did not describe evidence of direct podocyte infection. Most recently, Popik et al., described BKV cytopathology and lytic replication in undifferentiated and differentiated podocytes in vitro, as demonstrated by high expression levels of VP1 total protein and mRNA post infection [34]. The authors also observed induction of CXCL10 and IFNβ transcriptional in BKV-infected podocytes that correlates with increased viral replication over the course of infection [34]. It is unclear if podocyte infection with BKV plays a direct role in BKVAN progression in vivo. Like mesangial cells, podocytes are immune responsive to BKV. Thus they may be able to clear BKV in vivo or avoid significant infection by the recruitment or of host factors that protect the cells against BKV infection, or by subverting those that enhance infection. 

### 4.5. Glomerular Endothelial Cells

Until recently, there was only one case report of BKV-related polyomavirus vasculopathy in a renal transplant patient [63]. In this study, Petrogiannis-Haliotis et al. describes a 52-year-old male patient who had developed ESRD after undergoing a cadaveric renal transplantation [63]. The patient suffered from BKV vasculopathy resulting from virus infection of vascular endothelial cells [63]. BKV antigen expression was detected in endothelial cells by immunohistochemistry in renal biopsies and BKV DNA was identified in an extract of frozen kidney tissue by polymerase-chain-reaction (PCR) using BKV-specific primers [63]. Ultrastructural analysis by electron microscopy revealed BKV-infected endothelial cells in both the transplanted and native kidneys, but immunoperoxidase staining did not detect any virus in the renal tubules [63]. Recent, in vitro studies by Popik et al., using primary human glomerular endothelial cells (GECs), revealed that GECs are highly permissive for BKV infection and lytic replication, as demonstrated by BKV cytopathology as well as high expression levels of the BKV LTAg and VP1 [34]. They also observed the induction of a IFNβ transcription gene in BKV-infected GECs that correlates with increased viral replication over a time course of infection [34]. The authors also observed varying levels of CXCL10 induction over a time course of infection. In a recent study by An et al., the authors also observed an induction of CXCL10 and IFNβ expression in BKV-infected human GECs, along with the activation of IRF3 and STAT1 [64]. Findings from these studies support the notion that GECs can mount an immune protective response to BKV infection and may act as an immune barrier to BKV infection in vivo. These immune protective factors or receptors that may be suppressed or downregulated in vitro could render GECs more permissive for BKV infection and explain the rare occurrence GECs infection in vivo. Figure 2 shows a hypothetical model of cell types and routes of BKV dissemination in the proximal and distal compartments of the human glomerulus. 

## 5. The Potential Role of Glomerular Parenchymal Cells (GVU cells) in BKV Latency/Reactivation and BKVAN 

**Figure 3:**
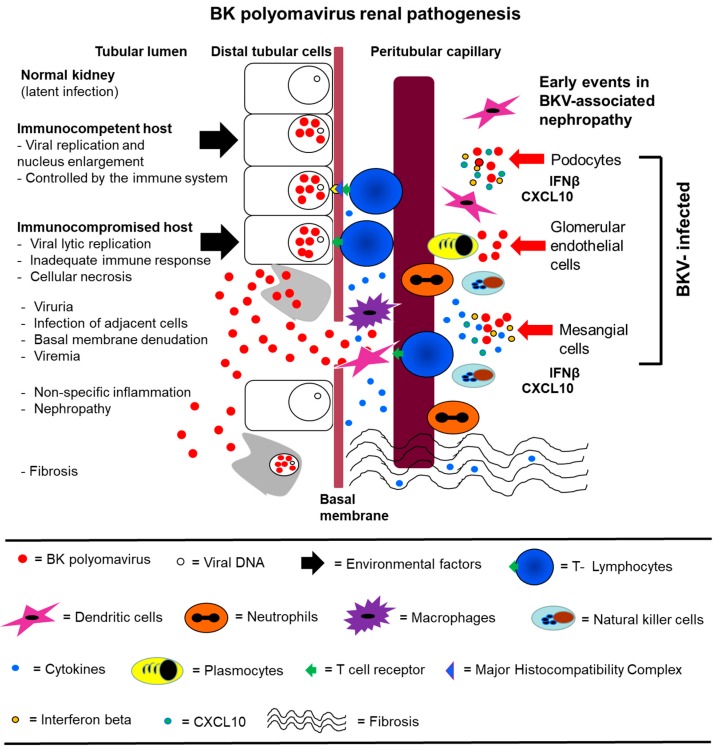
A hypothetical model for BKV pathogenesis that include infection of GVU cells. (**Left**) BKV (red spheres) infection of a normal kidney exists in the latent state in distal tubular epithelial cells. Initial BKV replication is then controlled by the immune system in an immunocompetent host. In the immunocompromised host, viral replication is not controlled, leading to extensive lytic replication and cellular necrosis. Viruria and viremia ensue, and the infection spreads to adjacent cells and the basement membrane is compromised. There is extensive inflammation and recruitment of immune effector cells. Nephropathy occurs followed by interstitial fibrosis. (**Right**) GVU cells are initially infected by BKV which leads to the induction of IFNβ and CXCL10. CXCL10 plays a role in the recruitment of immune effector cells that contribute to inflammation. The induction of IFNβ and IFNβ pathways may protect some GVU cells from BKV infection by establishing an immune barrier and promoting viral clearance. Model of BKV renal pathogenesis (modified with permission from Lamarche et al., BK polyomavirus and the transplanted kidney: Immunopathology and Therapeutic Approaches *Transplantation* 2016).

GVU cells have all been shown to be permissive for BKV infection in vitro. However, in vivo infection of GVU cells is rare in patients with BKVAN, possibly due to differential receptor expression, down regulation of the primary receptor, or induction of an antiviral host factors that promote viral clearance. Immunosuppression and concomitant suppression of T-cell immune surveillance trigger BKV reactivation from latency in renal transplant patients, subsequently leading to high levels of viral replication in the tubular epithelium. As a result, these patients develop BKV-associated nephropathy. The resulting denudation of the basement membrane, followed by robust viremia resulting in uncontrolled inflammation, can lead to nephropathy and fibrosis (Figure 3.) I propose that podocytes and mesangial cells may aid in the early phase recruitment of immune effector cells via the induction CXCL10 (Figure 3). In addition, the induction of IFNβ in these cells may be protective against BKV infection, due to the cytokine’s antiviral and anti-proliferative effects [64,65]. Taken together, it is likely that GVU cells serve as potential latent BKV reservoirs that contribute to early events in BKV reactivation. Comprehensive studies that examine temporal events and early stages of BKV infection are needed to identify target cells in renal biopsies prior to development of BKVAN. Examination of renal biopsies to detect both BKV antigen and DNA would provide clues to the role that GVU cells play in BKVAN with respect to viral latency and reactivation. 

## 6. Rare Appearance of BKV in Glomerular Parenchymal Cells in Renal Biopsies of Patients with BKVAN

The role of BKV replication in glomerular parenchymal cells, as well as their contribution to viral latency, reactivation, and renal pathology associated with BKVAN, has been largely unexplored. Tubular epithelial cells may represent a selected cell type for BKV infection, because of their inability to mount an immune response against the virus. They also support high levels of viral replication. In other words, the tubular epithelium provides a functional microenvironment for BKV reactivation and provides an ideal site as a BKV reservoir for infection. GVU cells have all been shown to exhibit IFNβ induction in vitro, which may mount an antiviral response in uninfected cells during early stages of BKV reactivation. Elucidation of specific viral and host factor interactions required for BKV latency is warranted. Findings from these studies may explain why BKV infection is rarely detected in in GVU cells in vivo. 

## 7. Discussion

BKV infection and reactivation following immunosuppression are important causes of renal allograft dysfunction and graft loss. These conditions eventually lead to BKVAN. Understanding the role of both proximal and distal glomerular cells in BKVAN progression will allow investigators to determine the pathogenic mechanisms involved in BKV trafficking and infection profiles, as well as additional viral reservoirs, and conditions required for the establishment of viral latency and reactivation. Future studies may help to advance the development of novel strategies to protect targeted cells in the glomerulus from BKV infection before and after immunosuppression. These studies may also contribute to novel strategies for early diagnosis and subsequent early interventions that aid in the recovery of renal function.
